# Sodium butyrate alleviates deoxynivalenol-induced hepatic cholesterol metabolic dysfunction via RORγ-mediated histone acetylation modification in weaning piglets

**DOI:** 10.1186/s40104-022-00793-1

**Published:** 2022-12-23

**Authors:** Qiufang Zong, Huan Qu, Yahui Zhao, Haoyu Liu, Shenglong Wu, Shuai Wang, Wenbin Bao, Demin Cai

**Affiliations:** 1 College of Animal Science and Technology, Yangzhou University, Yangzhou, 225009 PR China; 2 Joint International Research Laboratory of Agriculture & Agri-Product Safety, Yangzhou University, Yangzhou, 225009 PR China; 3 Department of Animal Nutrition and Feed Science, College of Animal Science and Technology, Huazhong Agricultural University, Wuhan, 430070 PR China

**Keywords:** Cholesterol biosynthesis, DON, Histone acetylation, RORγ, Sodium butyrate

## Abstract

**Background:**

Cholesterol is an essential component of lipid rafts in cell plasma membrane, which exerts a hepatoprotective role against mycotoxin exposure in pigs, and cholesterol metabolism is vulnerable to epigenetic histone acetylation. Therefore, our present study aimed to investigate whether a histone deacetylase inhibitor (sodium butyrate [NaBu]) could protect the porcine liver from deoxynivalenol (DON) exposure by modulating cholesterol metabolism. Herein, we randomly divided 28 pigs into four groups, which were fed an uncontaminated basal diet, contaminated diet (4 mg DON/kg), uncontaminated diet supplemented with 0.2% NaBu or 4 mg/kg DON contaminated diet (4 mg DON/kg) supplemented with 0.2% NaBu for 28 d.

**Results:**

We found that the serum alanine transaminase (ALT), aspartate transaminase (AST), and alkaline phosphatase (ALP) were all increased in pigs exposed to DON, indicative of significant liver injury. Furthermore, the cholesterol content in the serum of DON-exposed pigs was significantly reduced, compared to the healthy Vehicle group. Transcriptome analysis of porcine liver tissues revealed that the cholesterol homeostasis pathway was highly enriched due to DON exposure. In which we validated by qRT-PCR and western blotting that the cholesterol program was markedly activated. Importantly, NaBu effectively restored parameters associated with liver injury, along with the cholesterol content and the expression of key genes involved in the cholesterol biosynthesis pathway. Mechanistically, we performed a ChIP-seq analysis of H3K27ac and showed that NaBu strongly diminished DON-increased H3K27ac genome-wide enrichment. We further validated that the elevated H3K27ac and H3K9ac occupancies on cholesterol biosynthesis genes were both decreased by NaBu, as determined by ChIP-qPCR analysis. Notably, nuclear receptor RORγ, a novel regulator of cholesterol biosynthesis, was found in the hyperacetylated regions. Again, a remarkable increase of RORγ at both mRNA and protein levels in DON-exposed porcine livers was drastically reduced by NaBu. Consistent with RORγ expression, NaBu also hindered RORγ transcriptional binding enrichments on these activated cholesterol biosynthesis genes like *HMGCR*, *SQLE,* and *DHCR24*. Furthermore, we conducted an in vitro luciferase reporter assay to verify that porcine RORγ directly bonds to the promoters of the above target genes.

**Conclusions:**

Collectively, our results demonstrate the utility of the natural product NaBu as a potential anti-mycotoxin nutritional strategy for regulating cholesterol metabolism via RORγ-mediated histone acetylation modification.

## Background

Mycotoxins are secondary metabolites produced by filamentous fungi and can be ingested by animals and humans by accident with a high risk of acute or chronic toxicity [1, 2]. Worldwide, approximately 60%–80% of cereals are contaminated by various molds every year, thus directly threatening livestock and poultry production [3]. Deoxynivalenol (DON) is one of the most widely distributed mycotoxins. Its effect is often dose- and/or exposure time dependent, and can cause severe damage to the liver, gastrointestinal tract, and other metabolic and immunity-related organs [4]. As the central metabolic site, liver is responsible for detoxification following mycotoxin exposure. Given the fact that DON can hardly be removed 100% by the approaches of physical elimination, chemical degradation, and biodegradation, a small amount of accumulation can still cause liver damage in pigs [5]. Therefore, it is a burning question to explore the novel strategy against DON-induced toxicity in the porcine liver via endogenous approaches.

Notably, as the liquid-ordered microdomains in the plasma membrane, lipid rafts play a hepato-protective role by maintaining cellular membrane integrity to diminish mycotoxin-induced hepatocyte damage [6]. Owing to that lipid rafts are composed of cholesterol and sphingolipids, maintenance of cholesterol homeostasis is of great importance to ameliorate mycotoxin-induced liver injury as documented [7]. In contrast, mycotoxin exposure can elicit altered cholesterol production and dynamic balance, likely via the modulation of genes involved in cholesterol metabolic processes [8]. This suggests these genes are critical players enrolled in the prevention of mycotoxin damage. Notably, studies have revealed that cholesterol metabolic genes are susceptible to epigenetic regulation including DNA/RNA methylation and histone modifications [9]. Especially in the liver of pigs, histone acetylation exerts pivotal actions in the cholesterol metabolic gene programming [10]. Despite an increasing number of histone deacetylase (HDAC) modulators being widely studied to control acetylated events, sodium butyrate (NaBu), a natural and endogenous HDAC inhibitor, has attracted more attention in the last decade [11]. As expected, NaBu has been proven to mediate multiple biological reactions, including cholesterol metabolism, possibly via the regulation of histone acetylation [12]. Importantly, NaBu is thought to ameliorate the liver lesions triggered by DON in piglets [13]. However, the underlying mechanisms have not been elucidated.

Tremendous research progress has indicated that various nuclear receptors (NRs), including RORα/γ, REV-ERBα/β, LXRs, and PPARs, work as transcription factors (TFs) and play a cardinal role in controlling cholesterol metabolism by recruiting histone marks and co-factors [14, 15]. Notably, among multiple NRs, RORγ has been found to specifically regulate cholesterol biosynthesis over the typical TF SREBP2 in porcine liver organoids. RORγ activation by agonists or ectopic expression upregulates cholesterol biosynthetic gene expression via the hyper-enriched histone active mark H3K27ac [10]. It is elegantly proven that RORγ directly binds to the genes involved in cholesterol biosynthesis using a genome-wide ChIP-seq analysis, and facilitates the histones’ acetylation to enhance the cholesterol biosynthesis rate [16]. It is worth mentioning that RORγ is one of the crucial drivers of this process in newborn piglets, in close association with mycotoxin-induced hepatic cholesterol re-programming and histone acetylation modifications containing H3K27ac [8]. In spite of that the expression and function of part of the NR members are found to be modified by NaBu, the direct evidence of the crosstalk between RORγ and NaBu has not yet been reported. Thus, we herein hypothesized that RORγ acts as a vital player enrolled in NaBu-alleviated liver injury in DON-exposed piglets via epigenetic regulation of cholesterol metabolism programming by histone acetylation.

## Methods

Given that piglets are vulnerable to DON exposure, we generated a liver injury condition in piglets by dietary DON administration and then studied the hepatoprotective effects of NaBu. Cholesterol homeostasis was evaluated based on the total concentration in the liver, as well as using transcriptome analysis of hepatic cholesterol metabolism gene expression profiles. H3K27ac ChIP-seq analysis was used to examine genome-wide histone modification to verify RORγ actions on cholesterol metabolism gene programming. RORγ transcriptional/translational expression, and in vitro luciferase reporter assays were used to further validate the RORγ targetable functions via directly binding fashions. Our present results probe the potential of NaBu as a bona fide agent for anti-mycotoxin through RORγ-programmed epigenetic mechanisms of cholesterol metabolic genes.

### Experimental animals and sampling

**Fig. 1:**
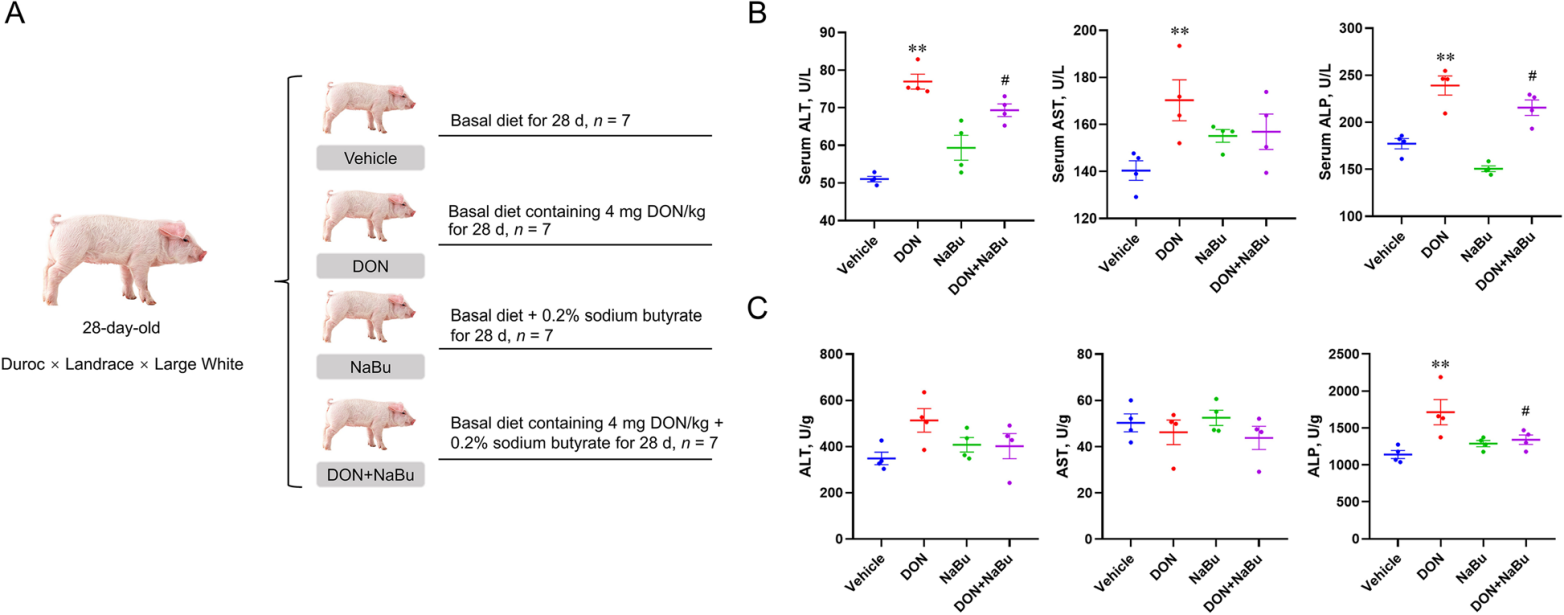
Sodium butyrate alleviates DON-induced liver injury in piglets. **A** Schematic illustration of the experimental design (*n* = 7 per group). **B** AST, ALT, and ALP concentrations in serum. **C** AST, ALT, and ALP concentrations in the liver. Data are presented as the means ± SEM of at least three independent experiments. ^*^P < 0.05, ^**^P < 0.01. ^*^Compared with the Vehicle group; ^#^compared with the DON group. One-way ANOVA was used

The animal study proposal was approved by the Institutional Animal Care and Use Committee (IACUC) of the Yangzhou University Animal Experiments Ethics Committee (permit number: SYXK (Su) IACUC 2012–0029). All experiments were conducted in accordance with the relevant guidelines and regulations. Twenty-eight barrows (28-day-old) were housed in an animal center, and randomly assigned to the following four groups: Vehicle (basal diet), DON (basal diet containing 4 mg DON/kg), NaBu (basal diet supplemented with 0.2% NaBu), and DON+NaBu (basal diet containing 4 mg DON/kg and 0.2% NaBu) (Fig. 1A). All of the pigs had ad libitum access to feed and water. The basal diet was formulated to satisfy the nutrient requirements of swine [17]. The DON feed was kindly provided by Huazhong Agricultural University [18], and the DON content was determined using an AgraQuant DON ELISA kit (Romer Labs, Singapore) according to the manufacturer’s protocol. NaBu was purchased from Sigma-Aldrich (St. Louis, MO, USA) and added to the basal diet to form a NaBu content of 0.2%. After 28 d of feeding, blood samples were taken from all pigs via puncture of the jugular vein and centrifuged at 4000 r/min for 20 min to obtain serum. Thereafter pigs were euthanized with sodium pentobarbital, and liver samples were collected and immediately frozen in liquid nitrogen and preserved at − 80 °C.

### Analysis of biochemical parameters

The concentrations of serum AST, ALT, ALP, CHO (cholesterol), TG (triglyceride), and TBA (total bile acids) were determined using commercial assay kits (Alovet, Suzhou, China) according to the manufacturer’s protocols using ALOVSION LIC200 (Alovet, Suzhou, China). Moreover, the protein of the liver was extracted, and the protein supernatant was collected for the determination of the biochemical index.

### Quantitative real-time PCR (qRT-PCR)

Total RNA was isolated using TRIzol reagent (Takara, Dalian, China). After synthesis from the RNA using HiScript® II Q Select RT SuperMix (Vazyme, Nanjing, China), cDNA was subjected to qRT-PCR amplification using a StepOne Plus Real-Time Quantitative PCR System (Applied Biosystems, CA, USA). The primers were synthesized by Bioengineering (Shanghai, China). Primer information is shown in Table 1. The relative level of each transcript was normalized to that of *GAPDH* and analysed according to the 2^−ΔΔCt^ method [19].

**Table 1:** Real-time PCR primer sequences

Genes	Primer sequences (5′→3′)
*ACAT2*	F: TAATGATGGTGCTGCTGCTGTGG R: GCTTGCTTTATTGCCGGGATTGG
*HMGCS1*	F: AAGCACAGCCACCGAGCATATTC R: ACCATCCCACCCCACACTGAAG
*HMGCR*	F: TGTGATTGGAGTTGGCACCATGTC R: ACACGCAAGCTGGGAAGAAAGTC
*MVK*	F: GTTGTCTCAAGTCCTGCTGGTGTC R: AGGCTCACTTTCCCACTGTTGTG
*PMVK*	F: GGTGGATGATGCTGAGTCAGAGTG R: GTGCTGCTCATCTCCGTGGTTC
*MVD*	F: GCCACCTGCTTGGACACCTTC R: GGCGAAGATCACGGCGTTGG
*FDFT1*	F: GCGTCCACCCTCCTCACTCC R: CCCACACAGCCAGAGCCAAAG
*SQLE*	F: TGTGGACCTTTCTCGGCATTGC R: TAGCGACAGCGGTAGGACAGC
*LSS*	F: GAGGACCCGCTGGTCCA R: CCACACTGTTCCTGTGCGC
*NSDHL*	F: TTTGTGATCGGGAACGGGAAGAAC R: TTCGTCATTGGTGATGTGGAAGGC
*DHCR24*	F: CCTCTTCCTCCTGCCGCTCTC R: TGCCCTGCTCCTTCCATTCCC
*RORC*	F: CAATGGAAGTGGTGCTGGTCAGG R: GGGAGCGGGAGAAGTCAAAGATG
*GAPDH*	F: ACATCATCCCTGCTTCTACTGG R: CTCGGACGCCTGCTTCAC

### RNA-seq and GSEA

Total RNA was extracted from the livers of pigs in the Vehicle, DON, NaBu, and DON+NaBu groups and prepared for RNA-seq library construction. PCR products were purified using an AMPure XP system (Beckman Coulter, CA, USA), and libraries were validated using an Agilent Bioanalyzer 2100 system (Agilent Technologies, CA, USA). Sequencing was performed on an Illumina HiSeq 2000 sequencer at BGI Tech (Wuhan, China). Reads were aligned to the genome assembly Sscrofa11.1 using TopHat2. Normalized gene expression was calculated using the HTSeq program. Differential expression analyses of DON vs. Vehicle and DON+NaBu vs. DON were performed using DESeq of the R package. Genes with an adjusted *P* value < 0.05 and |log_2_(fold change)| > 1 were defined as differentially expressed. Gene set enrichment analysis (GSEA 4.1.0) was applied to rank differentially expressed genes and enrich the biological processes and pathways.

### Western blotting

Frozen liver tissue was lysed in RIPA buffer (Beyotime, Shanghai, China) containing protease inhibitors. After centrifugation at 12,000 × *g* for 15 min at 4 °C, the supernatant was obtained, and proteins were quantified using an Enhanced BCA Protein Assay Kit (Beyotime, Shanghai, China). Proteins were electrophoresed and electrotransferred onto PVDF membranes (Millipore, MA, USA). The membranes were blocked with 5% skimming milk at room temperature (RT) for 2 h and incubated with primary antibodies at 4 °C overnight. After washing 3 times with cold PBS and incubating with a secondary antibody at RT for 2 h, the membranes were visualised using an ECL detection reagent and a FluorChem FC3 system (ProteinSimple, CA, USA). The antibodies used are shown in Table 2.

**Table 2:** Western blotting antibodies

Antibody	Manufacturer	Catalogue number	Dilution
MVK	Santa-Cruz	sc-390669	1:1000
MVD	Santa-Cruz	sc-376975	1:1000
FDFT1	Santa-Cruz	sc-271602	1:1000
SQLE	Santa-Cruz	sc-271651	1:1000
HMGCS1	Santa-Cruz	sc-166763	1:1000
RORγ	Invitrogen	14-6988-82	1:1000
GAPDH	Proteintech	10494-1-AP	1:5000
Anti-Mouse IgG	HuaBio	HA1006	1:10000
Anti-Rabbit IgG	HuaBio	HA1001	1:10000

### ChIP-qPCR and ChIP-seq data analysis

Porcine livers from the Vehicle, DON, NaBu, and DON+NaBu groups were ground in liquid nitrogen and subjected to crosslinking in 1% formaldehyde for 6 min, followed by quenching with glycine for 6 min. After 3 washes with PBS, the pellets were collected by centrifugation and resuspended in lysis buffer (50 mmol/L HEPES-KOH, 140 mmol/L NaCl, 10% glycerol, 1 mmol/L EDTA, 0.25% Triton-X100 and 0.5% NP-40). The pellets were resuspended in washing buffer (10 mmol/L Tris, 1 mmol/L EDTA, 0.5 mmol/L EGTA and 200 mmol/L NaCl). The supernatants were discarded, and then the pellets were resuspended in a shearing buffer (0.1% SDS, 1 mmol/L EDTA and 10 mmol/L Tris-HCl) for further sonication using a Covaris M220 (MA, USA) according to the manufacturer’s recommendations. Crude chromatin fragments were precipitated using antibodies and Protein G beads. After washing and adding proteinase K and RNase A, purified ChIP DNA was collected for further ChIP-seq and ChIP-qPCR assays.

The antibodies used in the ChIP assay were anti-RORγ serum (generated by Covance), H3K9ac (Abcam, ab4441), and H3K27ac (Abcam, ab4729). Collected DNA was quantified using an Agilent Bioanalyzer 2100 for sequencing on an Illumina HiSeq 2000 Sequencer (BGI, Wuhan, China). Raw single-end sequencing data (ChIP-sequencing reads) were checked for quality using FastQC (v0.11.9). The data were then aligned to the Sscrofa11.1 reference genome using Bowtie 1.2.3, followed by peak calling using MACS2 (2.1.1). Uniquely mapped tag filtering and deduping were used for peak calling by model-based analysis for ChIP-seq (MACS; 2.1.0) to identify regions of ChIP-seq enrichment over the background. Normalized genome-wide signal-coverage tracks from raw read alignment files were built using MACS2 and bedTools (https://github.com/arq5x/bedtools2). ChIP-seq signals at enriched genomic regions (avgprofile and heatmap) were visualised using deepTools (https://deeptools.readthedocs.io/en/develop/index.html). Peaks located in the promoter, 5′-UTR, 3′-UTR, exons, introns, and intergenic regions were annotated. Differential peaks in variant groups were picked using IGV software. The primers were designed based on the sequences of the peaks. ChIP-qPCR assays were performed according to the manufacturer’s recommendations. The primers are shown in Table 3.

**Table 3:** ChIP-qPCR primer sequences

Gene	Primer sequences (5′→3′)
*HMGCR*	F: TCTGAAGAAGTTTAAGGGAA R: GCTTGGGTTGCTGTTG
*SQLE*	F: CAGGACTGGCTTCTTC R: ATGGGTTCGGCACTAG
*HSD17B7*	F: ATGCTTGATAGGGTTT R: CAGAGGGTGTCAGTTT
*DHCR24*	F: ATTGCTGTGGCGTAGA R: GGTGCTTCAGAGGAGG
*LDLR*	F: ACAGCCATAGCAATACA R: TCAATGAGGAGGTAGGT

### Vector constructs and dual-luciferase reporter assay

For cholesterol homeostasis gene vector construction, we synthesized fragments of the *HMGCR*, *SQLE,* and *DHCR24* promoters with or without mutation of the putative RORC binding sites. HMGCR-wt and HMGCR-mut vectors were constructed by inserting the synthesized fragments of the *HMGCR* promoters from Chr2: 84,370,135–84,370,376 (Sscrofa11.1) and the mutant form from AGGTCA to CCCAAC into the pGL3-basic luciferase reporter vector. Similarly, SQLE-wt and SQLE-mut vectors were constructed by inserting the synthesized fragments of the *SQLE* promoters from Chr4: 14,684,789–14,685,079 (Sscrofa11.1) and the mutant form from TGACCT to GTTGGG into the pGL3-basic vector. DHCR24-wt and DHCR24-mut vectors were constructed by inserting the synthesized fragments of the *DHCR24* promoters from Chr6: 157,469,893–157,470,158 (Sscrofa11.1) and the mutant form from TGACCT to GTTGGG into the pGL3-basic luciferase reporter vector. After 293 T cells reached 70% confluence in 12-well plates, the wild types or mutant types of *HMGCR*, *SQLE,* and *DHCR24* genes were co-transfected with the *RORC* overexpression (RORC) vector. The renilla plasmid was co-transfected for normalization. Fluorescence activity was detected 36 h later using a dual-luciferase reporter system (Promega Corporation, WI, USA) according to the manufacturer’s instructions. Data were obtained from 3 independent experiments, each conducted in sextuplicate.

### Bioinformatic analysis using a clinical dataset

Data for mRNA expression-based liver cancer were downloaded from UCSC Xena datasets (https://xenabrowser.net/hub/). The data were then log2 transformed and quantile normalized before further analysis. Pearson’s correlation metric was computed between each gene using the ‘cor’ function in R, and the ‘ggplot2’ R package was used for further visualisation.

### Statistical analysis

All data were analysed using SPSS Statistics 21 software. Data are presented as the means ± SEM. Statistical analysis was performed using one-way ANOVA to compare the means. *P* < 0.05 was considered significant.

## Results

### Sodium butyrate alleviates DON-induced liver injury in piglets

To verify that DON can cause liver injury in piglets, the key parameters of liver injury including ALT, AST, and ALP levels in the serum and liver were analysed. As expected, elevated levels of ALT, AST, and ALP in the serum were observed in the DON-exposed piglets while these were recovered by NaBu treatment. Even though serum ALT and AST levels are increased in NaBu group, the effect is not significant and without statistical significance (Fig. 1B). Consistent with the results in serum, although the ALT and AST levels were not different among these four groups, NaBu markedly reduced the contents of ALP in the DON-exposed piglets compared to DON treatment alone (Fig. 1C).

### Sodium butyrate prevents DON-caused cholesterol metabolic abnormality

**Fig. 2:**
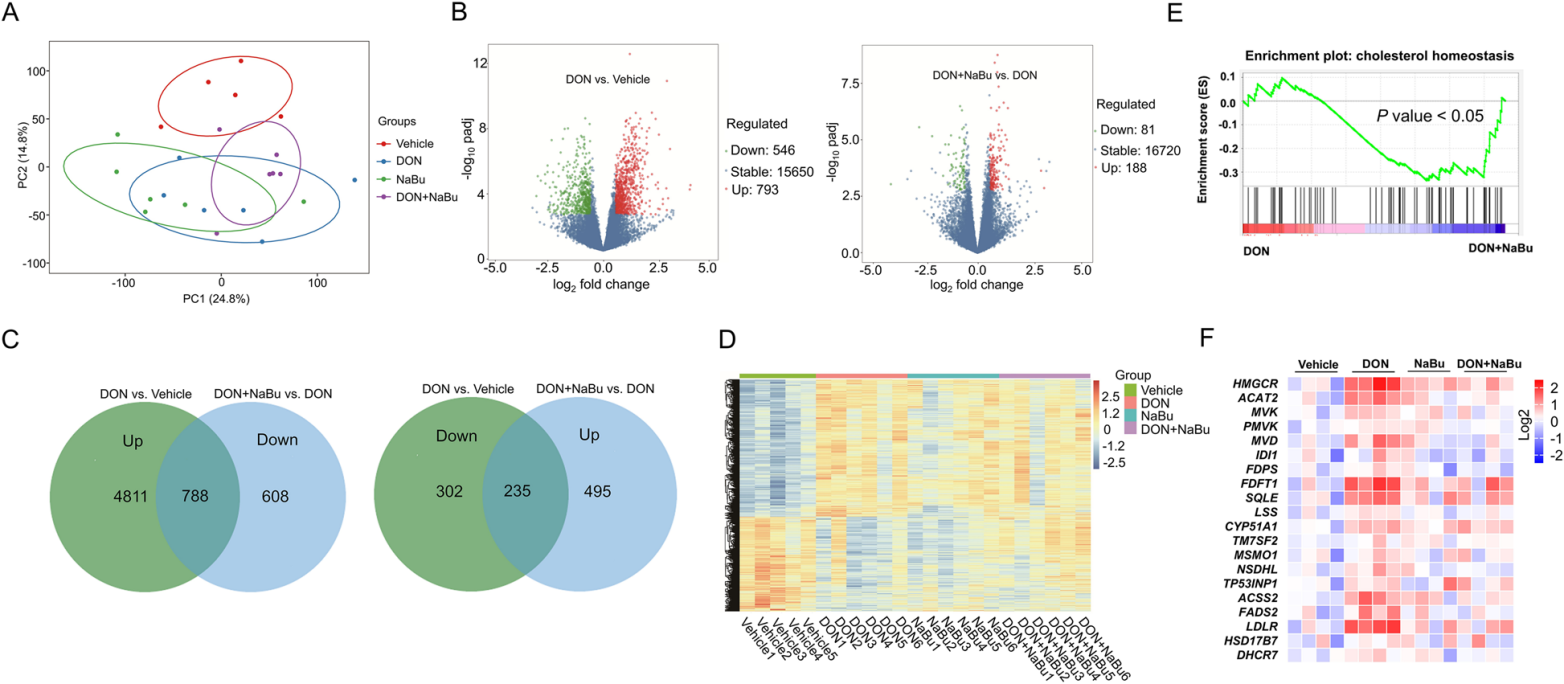
Transcriptome profile of porcine liver exposed to DON and NaBu supplementation. **A** Principal component analysis (PCA) plot for the four groups (Vehicle, DON, NaBu, DON+NaBu). **B** Volcano plots of differential expression profiles among the Vehicle, DON, and DON+NaBu groups. **C** Venn diagram of the number of genes with significantly differential expression (1.5-fold) changes. **D** A heatmap of cluster analysis of differential gene samples among the four groups. **E** GSEA plots depict the enrichment of genes down-regulated in the cholesterol homeostasis pathway in the porcine liver. **F** A heatmap of mRNA expression changes in cholesterol homeostasis genes, as determined by RNA-seq analysis in porcine liver

To explore the core transcriptional program in the liver of piglets upon DON contamination with or without NaBu, we performed RNA-seq analysis using liver tissues from the Vehicle, DON, NaBu, and DON+NaBu groups. We performed PCA by comparing gene expression levels using count data from the four groups. The results showed that single DON or NaBu treatment caused the obvious difference of the genes compared with Vehicle, while the combined DON with NaBu approach promoted the recovery of part of DON-altered genes (Fig. 2A). One thousand three hundred thirty-nine differentially expressed genes were identified between Vehicle and DON groups, among which were 793 up-regulated and 546 down-regulated genes by DON compared to that of Vehicle. Moreover, 269 differentially expressed genes were identified between DON and DON+NaBu groups, in which NaBu supplementation induced the upregulation of 188 and downregulation of 81 genes compared with DON treatment alone (Fig. 2B). Venn diagram manifested that the expression of 788 upregulated genes in the DON-treated piglets was inhibited by NaBu. In contrast, NaBu enhanced the expression of 235 genes in the porcine liver that was suppressed by DON exposure alone (Fig. 2C). Clustering of genes with expression significantly altered by DON (relative to Vehicle) showed a high degree of concordance in expression changes induced by NaBu (Fig. 2D). In line with these results, GSEA analysis revealed that the cholesterol homeostasis pathway was highly enriched and inhibited in the DON+NaBu groups versus DON treatment (Fig. 2E). Again, a pathway-focused analysis showed that the majority of cholesterol biosynthesis genes were significantly up-regulated by DON and then were decreased with the treatment of NaBu with DON (Fig. 2F).

**Fig. 3:**
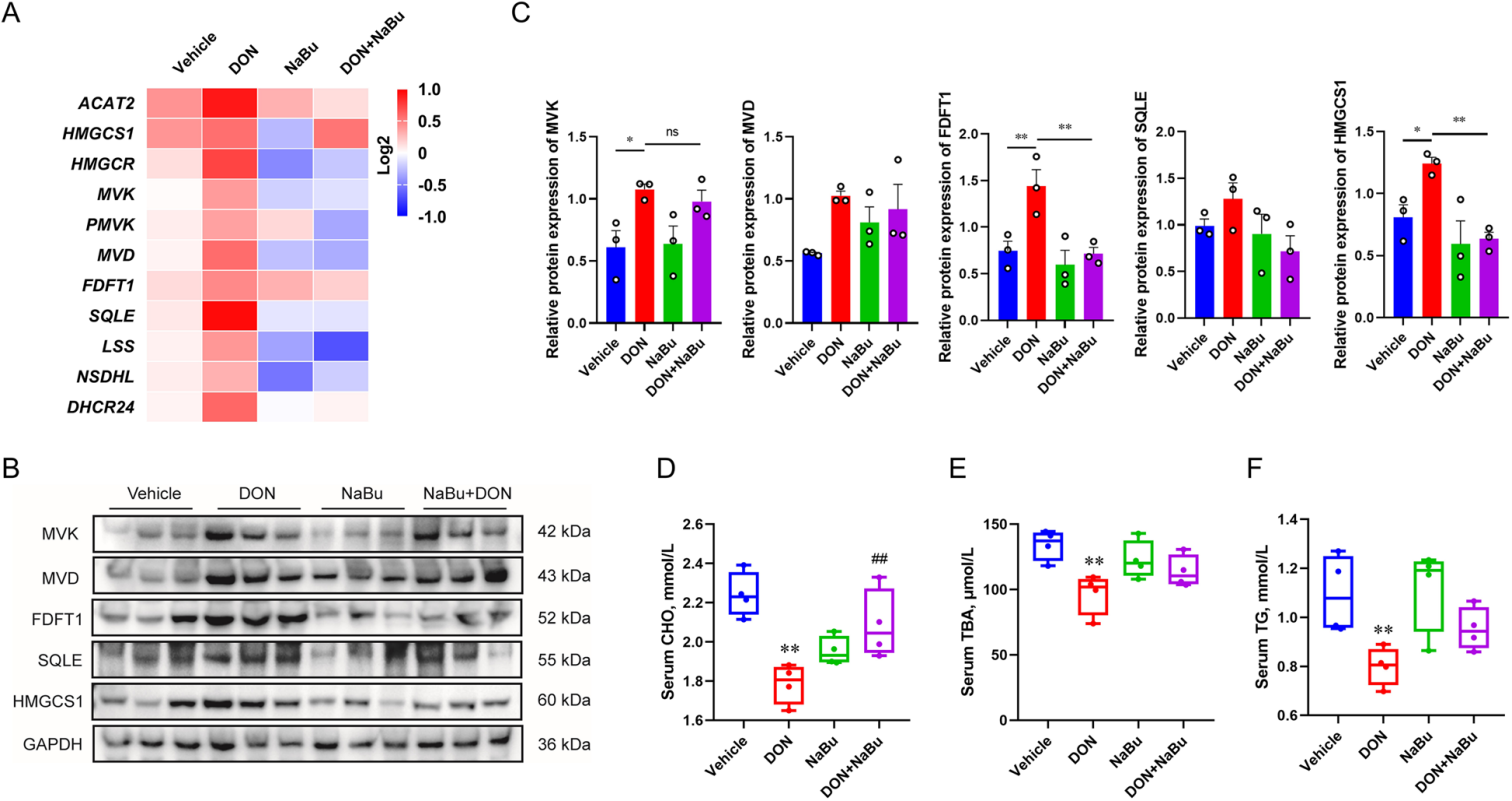
Sodium butyrate prevents DON-caused cholesterol metabolic abnormality. **A** A heatmap displays of fold-change (in log2) of gene mRNA levels in cholesterol homeostasis in porcine liver as analysed by qRT-PCR, *n* = 3. **B**, **C** Immunoblotting of proteins involved in the cholesterol homeostasis in porcine liver, normalized to *GAPDH* expression level. **D**-**F** (**D**) CHO, (**E**) TBA, and (**F**) TG contents in serum. Data are presented as the means ± SEM of at least three independent experiments. ^*^P < 0.05, ^**^P < 0.01, ns not significant. ^*^Compared with the Vehicle group; ^#^compared with the DON group. One-way ANOVA was used

To validate the RNA-seq results, the related mRNA expression was further measured by qRT-PCR. The expression of the key genes including *ACAT2*, *HMGCS1*, *HMGCR*, *MVK*, *PMVK*, *MVD*, *FDFT1*, *SQLE*, *LSS*, *NSDHL,* and *DHCR24* involved in cholesterol biosynthesis was significantly up-regulated by DON exposure, and was strongly inhibited in the group with the treatment of DON+NaBu compared to that of DON alone (Fig. 3A). Similarly, the immunoblotting results displayed that NaBu supplementation efficiently diminished the HMGCS1 and FDFT1 proteins expression to the comparable level of vehicle in the DON-exposed piglets, compared to that of single DON treatment (Fig. 3B and C). This was in association with the contents of CHO, TG, and TBA in the porcine serum with indicated treatment, in which the contents of the aforementioned index were significantly reduced in the DON-exposed group while increased in combination with NaBu (Fig. 3D-F).

### NaBu supplementation abolishes the enhanced genome-wide H3K27ac occupancy on cholesterol biosynthesis genes

**Fig. 4:**
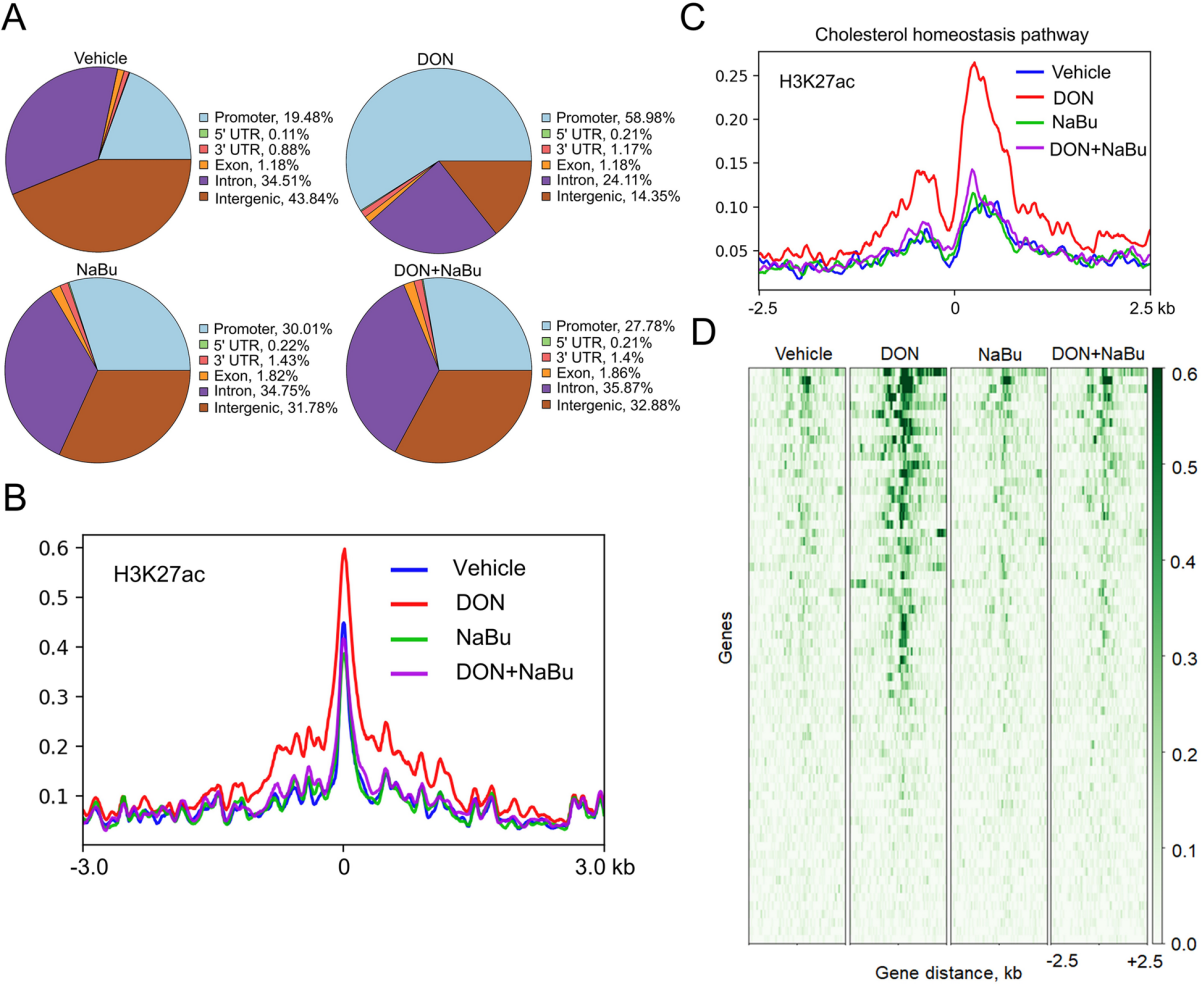
NaBu supplementation abolishes the enhanced genome-wide H3K27ac occupancy on cholesterol biosynthesis genes. **A** H3K27ac enrichment regions were localized within a 3-kb region of the promoter, exon, intron, UTR, and intergenic regions in porcine liver. **B** ChIP-seq profiles of H3K27ac signal intensity within ± 3-kb windows around the center of peak regions. **C** ChIP-seq profiles of H3K27ac binding on genes involved in the cholesterol biosynthesis pathway. **D** Heatmaps of ChIP-seq signal intensities of genes in the cholesterol biosynthesis pathway

To further explore the underlying mechanism by which NaBu regulates the cholesterol biosynthesis pathway, ChIP-seq analysis of histone acetylated mark H3K27ac was performed. We first demonstrated that the location of the binding sites was drastically shifted to promoter regions treated with DON, whereas NaBu markedly blunted the shift to display the similar pattern of the located binding regions to Vehicle (Fig. 4A). Moreover, DON exposure dramatically increased the genome-wide H3K27ac association with its targets, which was reduced by NaBu supplementation (Fig. 4B). Concomitant with the up-regulated transcripts involved in cholesterol metabolism, hyper-enriched H3K27ac was observed at the cholesterol metabolic gene loci in the DON-exposed piglets. Obviously, NaBu remarkedly decreased these relative enrichments as shown in the plots of Fig. 4C. Notably in the cholesterol biosynthetic program, as shown in the heat-map of H3K27ac signal intensity (Fig. 4D), NaBu supplementation caused the alterations of H3K27ac occupancies were in agreement with the mRNA levels of the aforementioned cholesterol biosynthesis pathway.

### Histone acetylated marks H3K27ac and H3K9ac contribute to NaBu-modulated key cholesterol biosynthetic genes

**Fig. 5:**
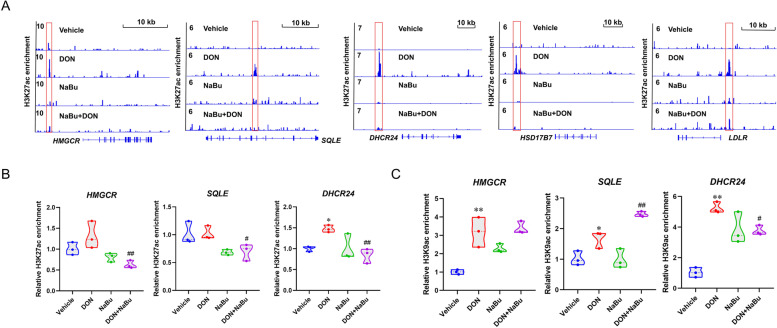
Histone acetylated marks H3K27ac and H3K9ac contribute to NaBu-modulated key cholesterol biosynthetic genes. **A** Visualisation of ChIP-seq signal of histone H3K27ac at indicated cholesterol homeostasis genes *HMGCR*, *SQLE*, *DHCR24*, *HSD17B7,* and *LDLR*. **B**, **C** ChIP-qPCR analysis of the relative enrichment of histone H3K27ac (**B**) and H3K9ac (**C**) at the *HMGCR*, *SQLE,* and *DHCR24* loci. Data are presented as the means ± SEM of at least three independent experiments. ^*^P < 0.05, ^**^P < 0.01. ^*^Compared with the Vehicle group; ^#^compared with the DON group. One-way ANOVA was used

Having revealed the crucial functions of histone acetylation in the regulation of the cholesterol metabolic pathway, we next examined which genes were susceptible to DON or NaBu exposure. Combined with the up-regulated transcriptional expression, H3K27ac enrichment showed a dramatic increment at the enhancers of *HMGCR*, *SQLE*, *HSD17B7*, *DHCR24* and *LDLR* in response to DON exposure, the significant loss caused by NaBu was also seen in the signal visualisation (Fig. 5A). Importantly, in addition to H3K27ac, we performed ChIP-qPCR analysis to validate and quantify the observations of histone acetylation. As shown in Fig. 5B and C, the cholesterol biosynthesis genes all displayed higher enrichments on the specific binding sites in the DON groups and these were diminished with NaBu treatment.

### RORγ is a key player involved in histone acetylation

**Fig. 6:**
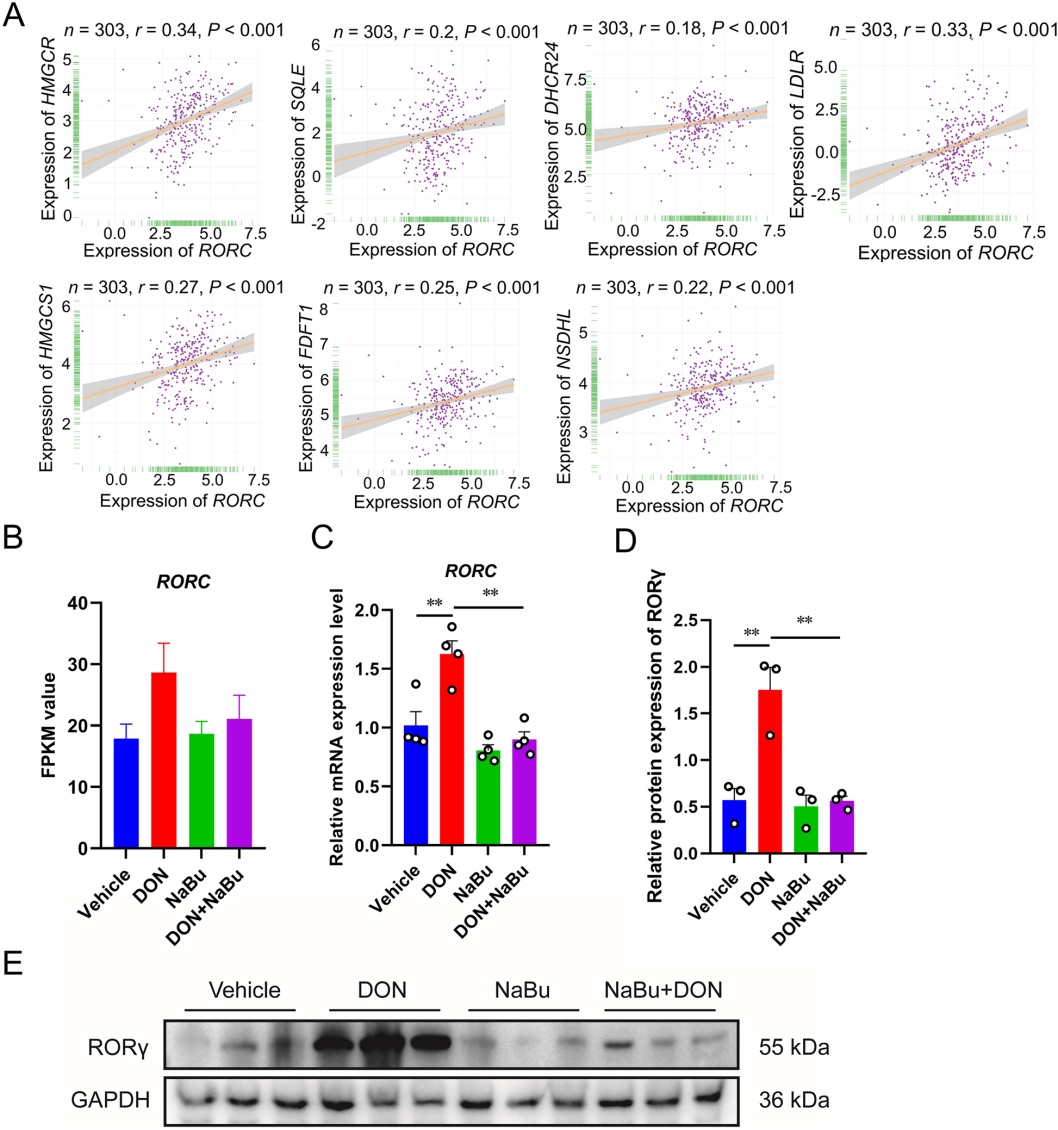
NaBu reverses the elevation of RORγ expression induced by DON exposure. **A** Correlation of transcriptional expression between *RORC* and cholesterol homeostasis genes, including *HMGCR*, *SQLE*, *DHCR24*, *LDLR*, *HMGCS1*, *FDFT1,* and *NSDHL*. **B** *RORC* gene mRNA expression determined by RNA-seq analysis in porcine liver. **C** *RORC* gene mRNA expression analysed by qRT-PCR in porcine liver. **D**, **E** Western blotting analysis of RORγ protein expression in the four groups. Data are presented as the means ± SEM of at least three independent experiments. ^*^P < 0.05, ^**^P < 0.01. One-way ANOVA was used

**Fig. 7:**
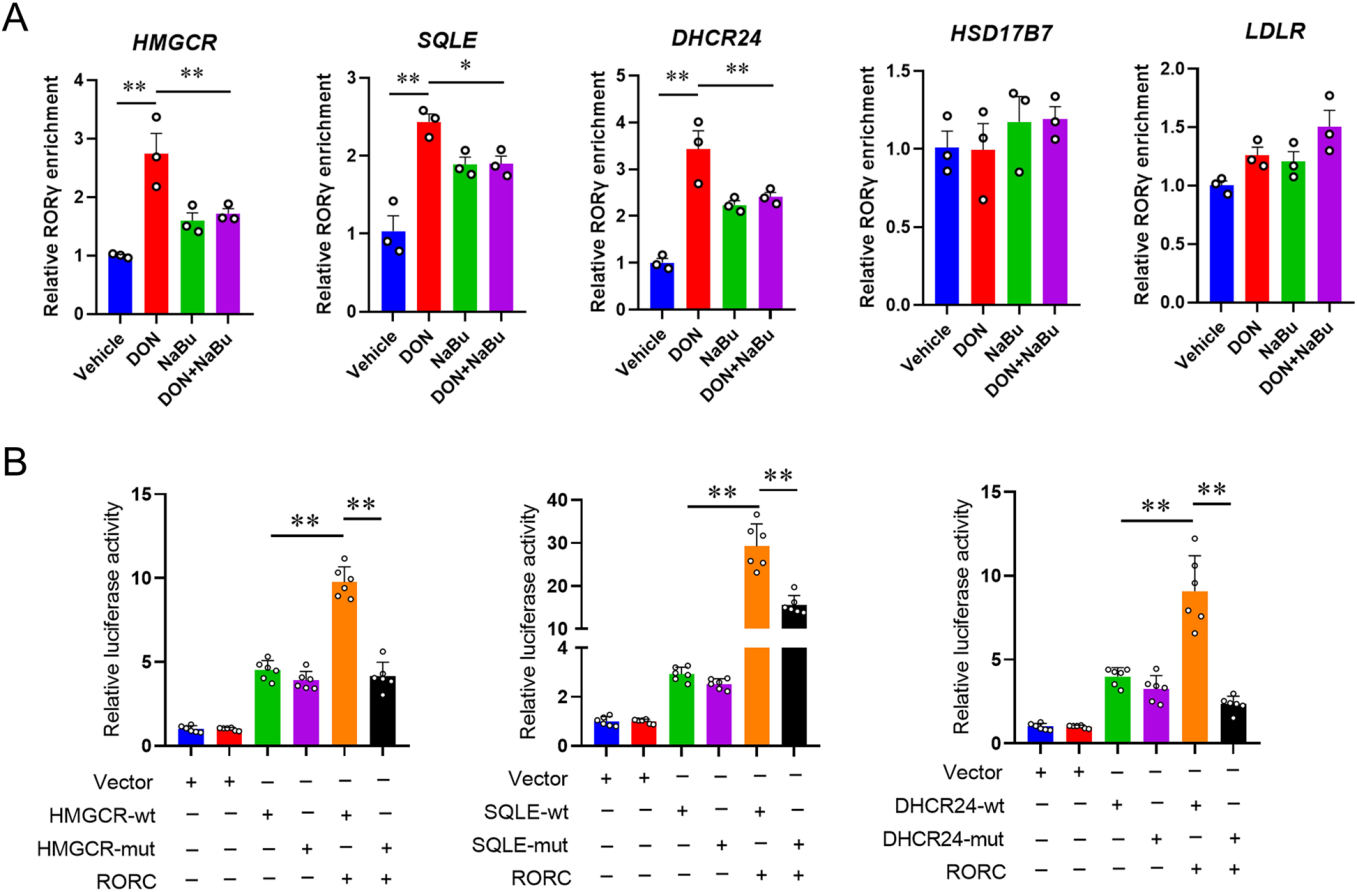
RORγ is a key player involved in histone acetylation. **A** ChIP-qPCR analysis of relative RORγ enrichment at genes as in (5A) following DON and NaBu treatment. **B** Promoter luciferase reporter activity changes of HMGCR, SQLE, and DHCR24 wild or mutant type activated with *RORC* overexpression. Data are presented as the means ± SEM of at least three independent experiments. ^*^P < 0.05, ^**^P < 0.01. One-way ANOVA was used

Nuclear receptor RORγ has been found to play a crucial role in the process of cholesterol biosynthesis [8]. Therefore, we aimed to further determine whether NaBu-regulated cholesterol biosynthesis is also reprogrammed by RORγ. Clinical dataset indicated a strongly positive correlation between the expression of *RORC* and *HMGCR* (*r* = 0.34, *P* < 0.001), *SQLE* (*r* = 0.2, *P* < 0.001), *DHCR24* (*r* = 0.18, *P* < 0.001), *LDLR* (*r* = 0.33, *P* < 0.001), *HMGCS1* (*r* = 0.27, *P* < 0.001), *FDFT1* (*r* = 0.25, *P* < 0.001) and *NSDHL* (*r* = 0.22, *P* < 0.001) (Fig. 6A). We then evaluated the FPKM value and mRNA expression of *RORC* gene, and the results revealed that the increased *RORC* expression in DON-exposed porcine livers was dramatically reduced by NaBu (Fig. 6B and C). In line with the mRNA expression results, NaBu supplementation alleviated DON-induced upregulation of RORγ protein expression (Fig. 6D and E). To determine whether RORγ is involved in histone modification in the present study, we performed ChIP-qPCR to detect the RORγ occupancy in the region of H3K27ac enrichment. As shown in Fig. 7A, consistent with the RORγ expression and histone acetylation modification results, NaBu hindered RORγ transcriptional binding enrichment on activated cholesterol biosynthesis genes. Moreover, in a dual-luciferase reporter assay, we found that promoters or enhancers of *HMGCR*, *SQLE,* and *DHCR24* genes were highly responsive to RORγ-dependent activation. However, the mutated type of the putative RORγ binding domain diminished the RORγ-mediated activation (Fig. 7B).

**Fig. 8:**
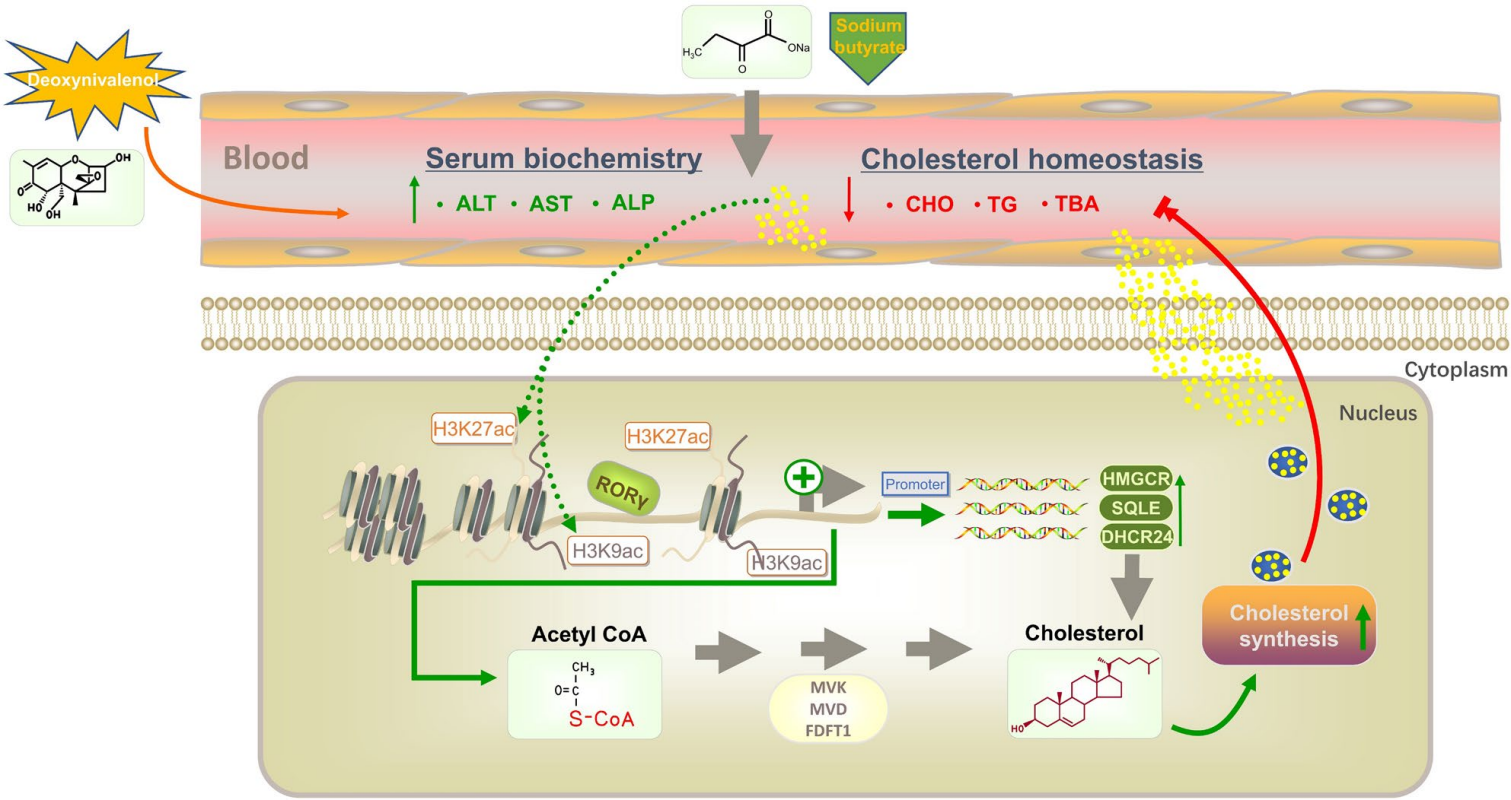
Schematic diagram of the mechanism by which sodium butyrate alleviates DON-induced disturbance of cholesterol homeostasis via RORγ-mediated histone acetylation modification in porcine liver

A graphical illustration of the mechanism by which NaBu alleviates DON-induced disturbances in cholesterol biosynthesis via RORγ-mediated histone acetylation is shown in Fig. 8. Our findings indicate that DON exposure causes liver injury, resulting in a decrease in cholesterol content in the serum and activation of the RORγ-mediated cholesterol biosynthesis pathway. When cholesterol levels in the body are elevated, RORγ expression and the continuous synthesis of cholesterol are in turn inhibited. This process is regulated by H3K27ac and H3K9ac acetylation.

## Discussion

Humans and animals are vulnerable to mycotoxin exposure because of the high toxicity and absorbability. In pigs, in particular, the absorption rate of DON is up to 66% as documented [20], which causes severe consequences in nearly all organs. Similar to previous findings of liver lesions induced by DON [21], here, we also found the obviously increased liver injury parameters in blood and liver, as ALT, AST and ALP were at pathological levels in the DON-treated piglets. It has been suggested that lipid dysfunction is closely associated with mycotoxin-induced liver lesions [22]. Notably, lipid rafts play cellular-protection roles in maintaining membrane properties and H^+^-ATPase activity against mycotoxin-induced structural failure of cell membrane by activating ceramide synthesis at the endoplasmic reticulum [23]. Lipid rafts are sub-domains of the plasma membrane and consist of cholesterol and sphingolipids, thus cholesterol homeostasis was our primary concern in the present study. As speculated, we observed that the cholesterol content in the serum of piglets was significantly reduced by DON exposure, suggesting that the hepatocyte damage was attributed to cholesterol depletion at cell membranes. Although the potential anti-mycotoxin action of NaBu has been suggested previously [13], we were still excited that NaBu strongly restored the cholesterol content and key parameters involved in liver injury. Notably, in the transcriptome analysis, the whole cholesterol metabolism pathway in the DON group is dramatically driven to the comparable level of Vehicle when NaBu was supplemented.

Given the classic function of NaBu in histone acetylation, we reasonably evaluated histone modifications on the key genes involved in cholesterol metabolism using our present model. Indeed, we have previously demonstrated that mycotoxins from commercial farms, including DON, ochratoxin, zearalenone, and aflatoxin B1 could markedly decrease cholesterol levels in pigs via epigenetic histone modification [8]. In this study, we found that the cholesterol biosynthesis pathway was upregulated in porcine livers when piglets were supplemented with DON alone. Indeed, hepatic cholesterol biosynthesis is typically negatively regulated via a signal derived from circular cholesterol loss. Because of the central site for cholesterol metabolism, this feedback loop facilitates the cholesterol balance among organs [24]. NaBu has been reported to modulate the expression of the genes involved in cholesterol biosynthesis and uptake [25]. Obviously, the increased expression of cholesterol biosynthesis genes in DON-treated piglets was down-regulated by NaBu supplementation. However, NaBu supplementation alone did not affect the cholesterol content and biosynthesis pathway, suggesting that a dose of 0.2% is most appropriate for anti-DON treatment in pigs, with no obvious side effects. Additional evidence of the safety of NaBu is the absence of an effect on ALT, AST, and ALP in the liver and serum compared with Vehicle group. Even though serum ALT and AST levels are increased in NaBu group. However, the effect is not significant and without statistical significance, and its levels are still within the normal physiological range [26–28]. Moreover, we also found that the genome-wide binding of histone acetylation mark H3K27ac was not changed by NaBu administration alone using ChIP-seq analysis. However, NaBu administration efficiently diminished DON-induced hyperacetylation both at the genome-wide level and of cholesterol synthesis genes. Indeed, NaBu, as a histone deacetylase inhibitor (HDACi), has been well documented to exert theoretical activity in epigenetically silenced genes by enhancing global histone acetylation [29]. Nevertheless, it is worth mentioning that HDACi has also been demonstrated to cause a similar amount or even more genes which are decreased than elevated [30]. Importantly, the majority of these alterations in gene expression with an even larger fraction triggered by HDACi are shown to be down-regulated [31, 32]. Combining our present results with previous reports, NaBu exhibited a couple of genomic characteristics on the DNA-bound histone acetylation status. Firstly, NaBu is more likely to create targets for histone deacetylation around the TSS regions while these genes are frequently down-regulated. In contrast, NaBu-induced genome-wide histone hyperacetylation continually occurs along the nuclear periphery. Secondly, the hyperacetylation of specific genes caused by NaBu is transient, whereas the histone acetylation status dynamically changes in a time-dependent manner. Additionally, NaBu would reset the structures and functions to regulate gene expression when initiating histone deacetylation [33].

Another novel finding in our present study is the NR RORγ action that mediates the NaBu-reprogrammed cholesterol biosynthesis transcript. Recent studies have provided a number of clues to control cholesterol metabolism in different scenarios by NRs including RORα/γ, REV-ERBα, and PPARα, directly or in combination with the classic cholesterol transcription factors SREBP2 and LXRs [14, 15, 34]. Importantly, RORγ has been demonstrated to drive cholesterol biosynthesis over SREBP2 by recruiting H3K27ac in a breast cancer cell line [16]. Notably, in a liver organoid model derived from piglets, RORγ was selected among a series of NRs as specifically modulating the cholesterol biosynthesis program [10]. In addition, we previously revealed that the hepatic protein content is positively correlated with the genome-wide binding enrichment of RORγ in mycotoxin-exposed piglets [8]. Herein, we further confirmed the potential regulatory effect of RORγ on the expression of cholesterol-related genes in pigs through a co-relationship analysis using clinical datasets and a luciferase reporter assay specifically targeting *HMGCR*, *SQLE* and *DHCR24*. In spite of that NaBu is suggested to activate NRs such as PPARs and VDR as a metabolite [35–37], the reports of its direct effects on RORγ are lacking. Interestingly, RORγt, a thymus-specific isoform of RORγ, was shown to be inhibited by NaBu in Th17 cells [38]. In agreement, NaBu supplementation dramatically suppressed RORγ expression at both the transcriptional and translational levels when exposed to DON. This inhibition was also shared at the chromatin binding occupancies that RORγ enrichments were similar to those of Vehicle in the pigs supplemented with DON along with NaBu. However, it is still unclear whether NaBu inactivates RORγ by directly binding to the ligand domain of RORγ. This is a limitation of the present study and needs to be explored in further investigations.

## Conclusions

Our study identified a novel mechanism in which RORγ targets cholesterol biosynthesis by regulating histone acetylation. These results contribute to the understanding of the role of RORγ in mycotoxin-induced liver injury in pigs. This study also illustrates a novel potential approach for mycotoxin poisoning prevention and intervention from the perspective of epigenetic modifications.

## Data Availability

The datasets analysed during the current study are available from the corresponding author upon request.

## Abbreviations

NaBu: Sodium butyrate
DON: Deoxynivalenol
ALT: Alanine transaminase
AST: Aspartate transaminase
ALP: Alkaline phosphatase
HDAC: Histone deacetylase
NRs: Nuclear receptors
TFs: Transcription factors
CHO: Cholesterol
TG: Triglyceride
TBA: Total bile acids
qRT-PCR: Quantitative real-time PCR
RT: Room temperature
HDACi: Histone deacetylase inhibitor
